# Identification and Functional Verification Reveals that miR-195 Inhibiting *THRSP* to Affect Fat Deposition in Xinyang Buffalo

**DOI:** 10.3389/fgene.2021.736441

**Published:** 2021-12-22

**Authors:** Shuzhe Wang, Cuili Pan, Xiaojie Ma, Chaoyun Yang, Lin Tang, Jieping Huang, Xuefeng Wei, Hui Li, Yun Ma

**Affiliations:** ^1^ Key Laboratory of Ruminant Molecular and Cellular Breeding of Ningxia Hui Autonomous Region, School of Agriculture, Ningxia University, Yinchuan, China; ^2^ College of Life Sciences, Xinyang Normal University, Xinyang, China; ^3^ State Key Laboratory for Conservation and Utilization of Subtropical Agro-Bioresources, College of Animal Science and Technology, Guangxi University, Nanning, China

**Keywords:** fat deposition, microRNA sequencing, bta-miR-195, Xinyang buffalo, THRSP

## Abstract

The buffalo population is extensive in China, but its meat quality is relatively inferior. Therefore, improving meat quality should be one of the breeding goals. microRNAs (miRNAs) play an essential regulatory role in the post-transcriptional expression of genes. Some studies have reported their function regulating genes related to fat deposition and adipocyte differentiation in cattle, but there is limited reports in buffalo. We performed small RNA transcriptome sequencing of Xinyang buffalo adipose tissue between calves and adults in this study. As a result, 282 mature miRNAs were significantly differentially expressed, and co-expression analysis showed that 454 miRNAs were significantly associated with developmental stages. Target gene identification, GO (gene ontology) annotation, and KEGG analysis of miRNAs showed that miR-195, miR-192, and miR-24-3p could target key genes for lipogenesis and thus regulate adipose deposition and differentiation. Among them, miR-195 was significantly upregulated in adipose tissue and induced adipocytes of adult buffaloes, and its overexpression significantly inhibited lipid accumulation in primary adipocytes. Dual-luciferase reporter gene analysis showed that miR-195 reduced the expression of thyroid hormone response protein (THRSP) by targeting its 3′ untranslated terminal region, suggesting that miR-195 may inhibit lipid accumulation in adipocytes by regulating *THRSP*. The results confirmed the reliability of predictive screening of miRNAs and provided theoretical support for buffalo fattening.

## Introduction

Fat formation is a highly complex process in mammals. Several studies have shown that miRNAs regulated (activation and inhibition) adipocyte differentiation and lipid accumulation (Remsburg et al., 2019). For example, *miR-21* was one of the earliest identified miRNAs which associated with adipogenesis and obesity, upregulated in white adipose tissue of obese subjects (Keller et al., 2011), promoted adipogenic differentiation (Kim et al., 2009), and facilitated lipogenesis (Mei et al., 2013). *miR-33a* and *miR-33b* played essential roles in controlling cholesterol homeostasis (Najafi-Shoushtari et al., 2010). Over-expression of *miR-33b* could reduce proliferation, impair differentiation, and decrease lipid droplets in precursor adipocytes (Price et al., 2016). *Let-7* could directly target *HMGA2* to inhibit the proliferation and differentiation of 3T3-L1 precursor adipocytes (Wei et al., 2014).

Historically, Asian wild buffalo were found throughout South and Southeast Asia but are classified as endangered, with less than 4,000 worldwide and probably less than 200 purebred Asian wild buffalo (Barker, 2014). Domestic buffalo are mainly distributed in Asian countries and are classified into *Bubalus bubalis* and *Bubalus carabanensis* based on morphology, behavior, and karyotype. The most recent data show that the *Bubalus bubalis* population is growing at a steady rate of 1,800,000 head/year, while the *Bubalus carabanensis* population is declining at a rate of 180,000 head/year (Zhang et al., 2020). It is due to the increase in the dairying of *Bubalus bubalis* and the decrease in the servitude of *Bubalus carabanensis* in agriculture. The Xinyang buffalo is characterized by largeness, robustness, sturdiness, and docility. The adults have a average body weight of more than 500 kg. However, traditional selection and breeding have led to inferior intra-muscular fat deposition ability and rough meat quality (Zuo and Liu, 2008). According to a previous study, the amount of Xinyang buffalo population was less than 150. Promoting the meat performance of Xinyang buffalo is a critical way to improve its breeding status and increase its quantity. Intramuscular fat (IMF), as a determinant of beef tenderness and succulence, is crucial for improving the quality of meat (Yamada et al., 2020). Given the comprehensive and vital roles of miRNAs in fat deposition and lipid accumulation, their expression profiles in humans, mice, pigs, cattle, and buffalo (Sun et al., 2014; Guller et al., 2015; Oclon et al., 2016) have recently been characterized in adipose tissue or adipose-associated cells, providing essential information for further research on fat deposition. The differences in miRNAs between buffalo adipose and muscle tissues have been explored in our previous study (Huang et al., 2019). However, details of the expression profiles and mechanisms of miRNAs in fat accumulation in buffalo are limited and require further investigation.

The growth and development of buffalo can be divided into the embryonic, juvenile, adolescence, and adulthood (Funston et al., 2010) period. The juvenile period lays the foundation for the production performance and physical appearance, and the adult buffalo has a strong body and fat deposition ability (Du et al., 2013). miRNAs in buffalo adipose tissue were sequenced and analyzed at two developmental stages (calf and adult buffalo) to screen for potential miRNAs regulating fat deposition. They were also subjected to weighted gene co-expression analysis to screen the miRNAs associated with growth and development. The conservatism of miRNA target genes between buffalo and cattle was compared, and the miRNA-mRNA interaction network related to adipocyte differentiation was mapped. Furthermore, the expression regulation mechanism of *miR-195* on key adipocyte differentiation genes was analyzed using a dual-luciferase reporter system and overexpression techniques. Our study lays the theoretical foundation for further revealing the mechanism of fat deposition in buffalo and improving the utility value of buffalo meat.

## Materials and Methods

### Ethics Statement

Animal experiments were conducted in accordance with the guidelines of the *Regulations for the Administration of Affairs Concerning Experimental Animals* (Ministry of Science and Technology, China, 2004). It is authorized by the Animal Ethics Committee of Ningxia University (permit number NXUC20190105).

### Animals and Sample Collection

The buffaloes were raised under the same dietary and environmental temperature conditions and given free water and food at the Xinyang buffalo farm (Xinyang, Henan, China). Subcutaneous adipose tissue was collected after slaughter and frozen immediately in liquid nitrogen. Three 6-month-old calves and three 30-month-old adult Xinyang buffaloes were slaughter to separate Rib eye upper back subcutaneous fat.

### Total RNA Extraction

Frozen tissue samples were homogenized in TRIzol reagent (Ambion, Life Technologies, NY), and total RNA was extracted from the supernatant according to the manufacturer’s protocol (Invitrogen, Carlsbad, CA). The quantity and integrity of RNA were confirmed using an Agilent 2100 Bioanalyzer (Agilent Technologies, Santa Clara, CA). Only RNA samples with integrity scores ≥7 were used for sequencing. Total RNA was stored at −80°C until further use.

### miRNA Bioinformatics Analysis

miRNAs were analyzed as follows: with aptamers removed by cutadapt software (version = V2.7) to obtain clean data; alignment analysis and filtering of remaining sequences using the RFam database (https://rfam.org/); miRDeep2 (version = 2.0.0.8) comparison of precursor sequences to identify miRNAs(Andrés-León et al., 2016).

Differentially expressed analysis of miRNAs was analyzed using the R package DEseq2 (version = 3.6.1) (Love et al., 2014). Target genes of DE miRNAs were predicted by TargetScan7.2 (http://www.targetscan.org/vert_72/), and the functional enrichment analysis of the target genes was performed by the DAVID website (https://david.ncifcrf.gov/) with a *p*-value ≤ 0.05 as the threshold.

To identify the critical modules and key miRNAs associated with adipose deposition, The R package (version = 4.0.3) WGCNA (Yao et al., 2019) was used.

### Validation of DE miRNA by qPCR

After removal of genomic DNA, total RNA was used for cDNA library construction, followed by reverse transcription at 37° 15 min, 85° 5 s; miRNAs were reverse transcribed by replacing the universal primer with a loop primer at 42° 15 min, 85° 5 s (Takara RR047A).

The qRT-PCR was performed using pre-denaturation for the 30s, PCR reaction for 90° 5 s, 60° 30 s, 45 cycles, followed by dissociation stage on an Applied Biosystems 7,500 Sequence Detection System (Takara RR0820A).

qRT-PCR was performed using SYBR Green PCR Master Mix on an Applied Biosystems 7,500 Sequence Detection System. The threshold cycle values indicated the quantity of the target gene in each sample. Negative controls used water as the template instead of cDNA. Three independent biological samples were used, and each run was performed in triplicate on plates. All related primers were designed using Primer 5.0 and synthesized by GeneScript (Supplementary Table S1).

### Cell Culture and Transfection

Subcutaneous adipose tissue from calves’ backs was collected for primary adipocyte culture according to the following procedure. After collection, the subcutaneous adipose tissue was immediately placed in 1% double antibodies (Hyclone, Scotland) in PBS (Hyclone, Scotland) and returned to the laboratory. The tissue pieces were washed three times in a sterile environment using PBS containing 1% double antibodies and separated into soybean-sized fat parts. The fat pieces were inoculated in 90 mm Petri dishes with inversion and incubated orthotopically after 6 h in DMEM (Hyclone, Scotland) high sugar medium with 10% fetal bovine serum (Gibico, Australia) and 1% double antibodies. Primary adipocytes can be freed after 4–5 days of culture.

The 293T cell line was maintained in this laboratory. All cells and cell lines were incubated and cultured in a jacketed water incubator at 37°C and 5% CO_2_. According to the manufacturer’s instructions, primary adipocytes of the third and fourth subculture generation were used for transfection by lipofectamine 3,000 (Thermo, United Staes). Add 10 μg/ml Insulin, 1 μM Dexamethasone, 0.5 mM 3-Isobutyl-1-methylxanthine, and 1 μM rosiglitazone for primary adipocyte induction (sigma, GRE).

### Dual-Luciferase Reporter Assay

The wild-type and mutant segments of thyroid hormone response protein (*THRSP*) 3′UTR containing the bta-miR-195 binding site were cloned by gamma two plasmids and used to construct dual-luciferase reporter gene vectors. The 293T cells were used, and the wild-type and mutant plasmids were co-transfected with bta-mir-195 agomiR.

### Statistical Analysis

qRT-PCR results were calculated using a 2^−∆∆Ct^ method. Statistical significance was tested using Graphpad Prism 7.0 software, and the Significant level was set as *p* < 0.05.

## Results

### Overview of miRNA Sequencing

Six miRNA libraries of buffalo subcutaneous adipose tissue from three calves and three adults were constructed and divided into two groups. A total of 10 GB of data was obtained. The size of libraries ranges from 10,702,344 to 30,835,129 raw reads. After filtering and aligning to mRNAs, RFam, and Repbase databases, 10,414,935–29,398,118 good reads were obtained. The libraries yielded 180,103–308,002 effective unique copies, accounting for 61.15–69.12% of the total sample (Supplementary Table S2). Base preference analysis of the miRNA sequencing (Supplementary Figures S1A,B) showed that the identity of the first base had an extremely high proportion of U (98%), which was consistent with the miRNA sequence characteristics.

Length distribution analysis demonstrated that miRNAs in the six libraries showed similar length patterns, varying from 18 nt to 26 nt, with 22 nt being the most common (Supplementary Figure S1C). Analysis of specific miRNA lengths was based on filtered datasets. Sequences with a length of 22 nt accounted for 44.13 ± 3.45% of the miRNAs, followed by 21 nt (14.96 ± 1.33%), and 20 nt (15.3 ± 1.64%) in the buffalo calf subcutaneous fat libraries. Sequences of 22 nt accounted for 44.13 ± 3.45%, followed by 22 nt (59.06 ± 7.09%) and 23 nt (6.1 ± 0.15%) in the adult buffalo libraries. These results were consistent with the characteristics of Dicer enzyme cleavage.

### Annotation and Identification of miRNAs

Genome annotation showed that 93.92 ± 1.43% of the total miRNAs could be mapped to the bovine reference genome (Supplementary Table S3). To obtain conserved miRNAs in subcutaneous fat tissue of buffalo, the ACGT101-miR tool was used to compare mapped reads in the reference genome with the known mature miRNAs in the miRase database. A total of 807 miRNAs (Supplementary Table S4) were obtained from the two adipose tissue developmental stages. Analysis of sequence source revealed that the number of miRNAs from exons or introns of coding genes was 13,977,471, accounting for 14% of the total reads. In contrast, rRNA, tRNA, snRNA, and other non-coding RNA accounted for 3.6%, indicating that the quality of the total RNA was favorable. In addition, miRNA expression varied across the two stages of growth, 665 miRNAs being obtained from adipose tissue of 6-month-old buffalo and 763 from 30-month-old buffalo. According to principal component analysis (PCA) (Supplementary Figure S2), the samples were well grouped and had high correlation coefficients. There were 618 overlap miRNAs in the two growth stages. These results showed that the expression of miRNAs was relatively high in the adipose tissue of adult buffalo.

### Identification of DE miRNAs

**FIGURE 1:**
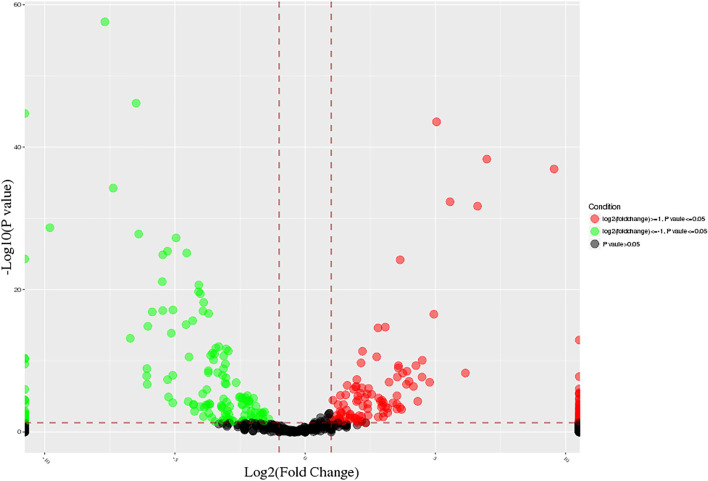
Volcano plot of all expressed miRNAs at different developmental stages. The “green” and “red” dots represent the down- and up-regulation miRNAs in the adult group, respectively.

DE miRNAs in calves and adult buffalo were identified using DEseq2 package in R with the screening standard set as | log2FC(fold change) | ≥ 1and adjusted *p*-value (FDR) ≤ 0.05. To elucidate the expression pattern of DE miRNAs, hierarchical clustering was conducted on the 618 overlap miRNAs. A total of 282 significantly differential expression miRNAs were screened from 807 miRNAs in the two groups, of which 148 were up-regulated, and 134 were down-regulated (Figure 1). These miRNAs showed significant differential expression during adipogenesis (*p* < 0.05), suggesting that these miRNAs are dynamically regulated during adipogenesis.

Furthermore, there were 282 differential expressed miRNAs across the two ages, of which 11 conserved miRNAs were specific to adult bovine adipose tissue, and 18 miRNAs were specific to calf adipose tissue.

### Identification of Hub miRNAs by WGCNA

WGCNA (Weighted Gene Co-expression Network Analysis) is used to identify gene modules with similar expression patterns. It dissects the association between gene sets and sample phenotypes or groups and identifies hub genes. In this study, we performed a WGCNA analysis of miRNAs and associated the identified modules with developmental stages to find hub miRNAs regulating adipose growth and development.

**FIGURE 2:**
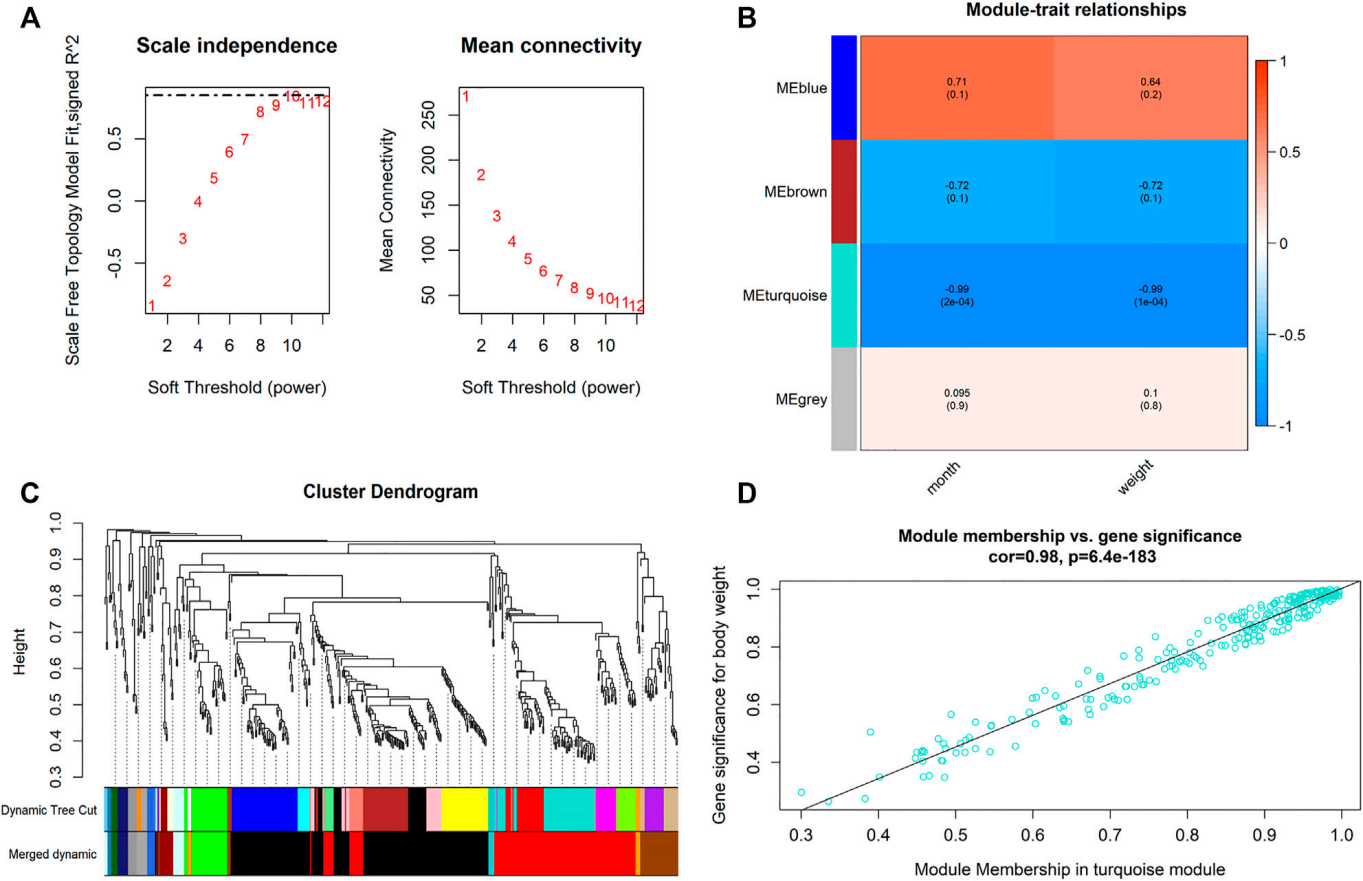
Main findings in the module-trait correlations analyses. **(A)** Analysis of the scale-free fit index for various soft-thresholding powers **(Left)** and analysis of the mean connectivity for various soft-thresholding powers **(Right)**; **(B)** Heatmap between the correlation between modules and weight (Each cell contained the correlation coefficient and corresponding *p* value; **(C)** Clustering dendrogram of differentially expressed genes related to Month and Weight in the deodunal tissues of six Xinyang buffalo; **(D)** The gene significance for weight in the turquoise module (One dot represents one gene in the turquoise module).

When 0.99 was used as the correlation coefficient threshold, the soft-thresholding power was selected as ten (Figure 2A). Through WGCNA analysis, four co-expression modules were constructed (Figures 2B,C). The turquoise module was comprising the most miRNAs (508 miRNAs), followed by the blue module (144 miRNAs), the brown module (88 miRNAs), and the grey module (15 miRNAs).

Module-trait correlations analyses were performed between modules and phenotypes after obtaining co-expressed modules (Figure 2D). Correlation analyses showed that turquoise modules were significantly associated with both weight and development stages (month) (*p* < 0.01). In this module, *bta-miR-129-5p*, *bta-miR-148a*, *bta-miR-195*, *bta-miR-93*, and *bta-miR-504* had high gene significance (GS > 0.2) with phenotype and showed high module-membership (MM) > 0.8, which were select as candidates for hub miRNAs.

### Enrichment Analysis of the Target Genes of miRNAs

**FIGURE 3:**
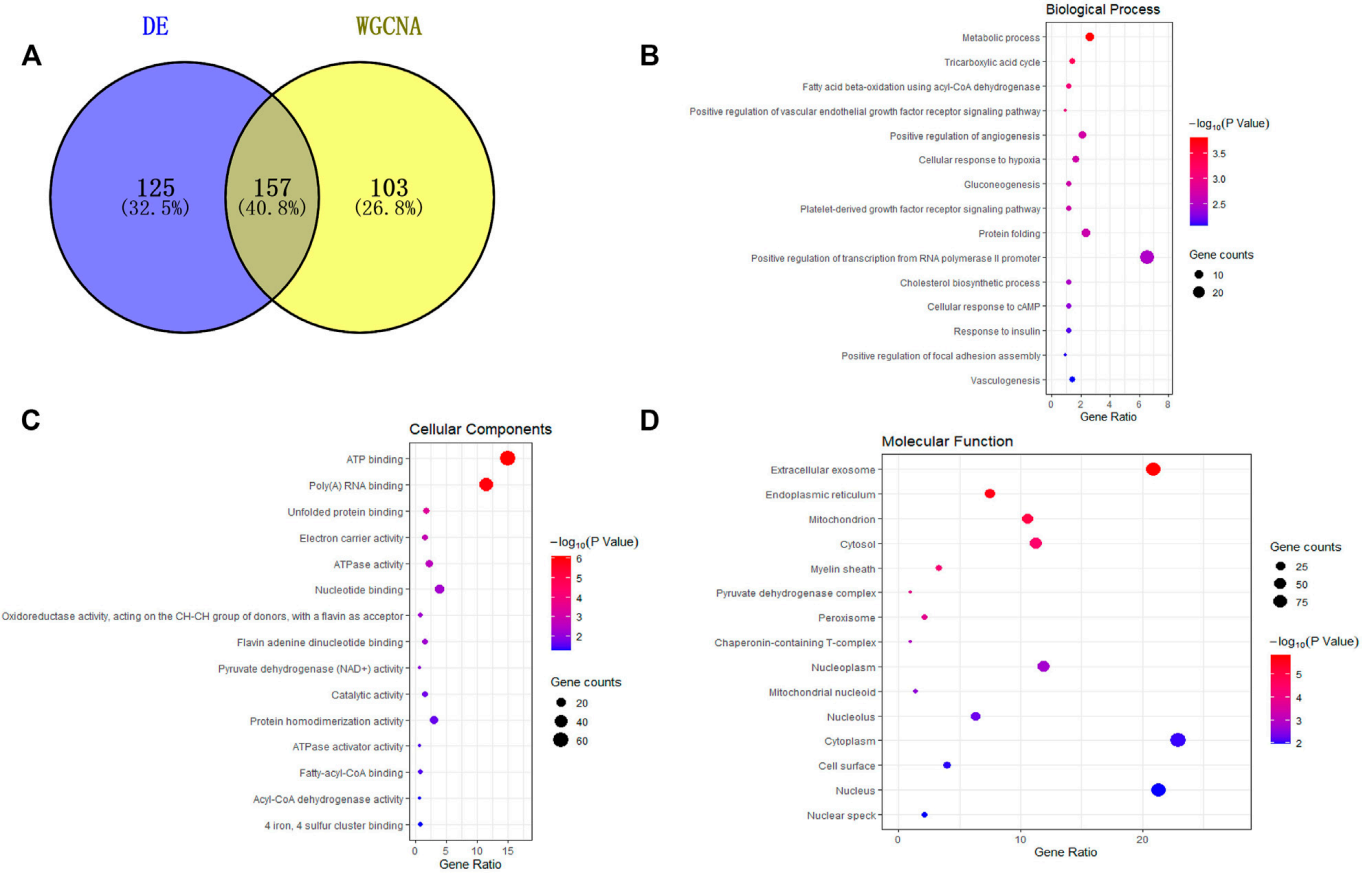
Functional analysis of miRNAs target genes. **(A)** Venn diagram of differentially expressed miRNAs and miRNAs in the turquoise module; **(B–D)** are the gene functional enrichment analysis of the target gene of miRNAs, and B**–**D are biological process, cellular components, and molecular function, respectively. The dot size represents gene amount enriched in a particular process, and the color represents significance.

A total of 157 miRNAs related to fat deposition were obtained by intersecting the miRNAs obtained from differential expression analysis and WGCNA analysis. (Figure 3A). Prediction of target genes showed these miRNAs could target 6,501 genes. The target genes were enriched in ten GO terms, including 83 biological process terms, 26 cellular component terms, and 35 molecular function terms. Of these, in total, 76 terms were significantly enriched (*p* < 0.05). Further analysis showed that the target genes were significantly enriched in translation, lipid transport, ATP synthesis coupled electron transport and endodermal cell differentiation (Figures 3B–D).

**FIGURE 4:**
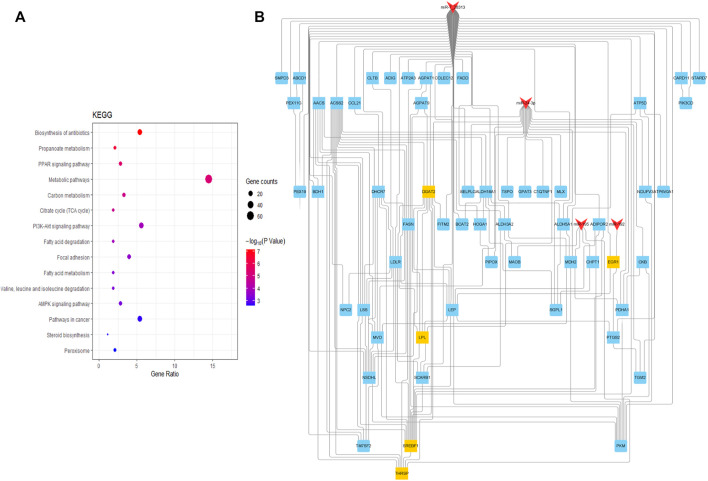
Diagram for KEGG analysis and PPI of the target gene. **(A)** Analysis of signaling pathways of miRNAs target genes. The dot size represents gene amount enriched, and the color represents significance; **(B)** PPI plot of the miRNAs-target gene and the red shape “V” represents miRNAs; the shape “squares” are target genes, yellow and blue squares are key target genes.

KEGG pathway enrichment analysis was performed to comprehensively understand the functions of the target genes at the two growth stages. The target genes of DE miRNAs were significantly enriched in 31 pathways associated with fatty acid (*p* < 0.05), including the fatty acid degradation, fatty acid metabolism, and PPAR signaling pathway (Supplementary Table S5) (Figure 4A).

TargetScan and RNAHybrid further predicted the target genes, and 98 target genes with extremely significant differential expression were selected for intersection with the above-predicted target genes. Finally, 103 credible candidate target genes were selected (Figure 4B). Four candidate target genes, *THRSP*, *EGR1*, *DGAT2*, *SREBF1*, and *LPL*, were screened and determined as hub genes related to fat deposition by interacting network analysis, which could be candidate genes for subsequent verification.

### Validation of DE miRNAs Using qRT-PCR

**FIGURE 5:**
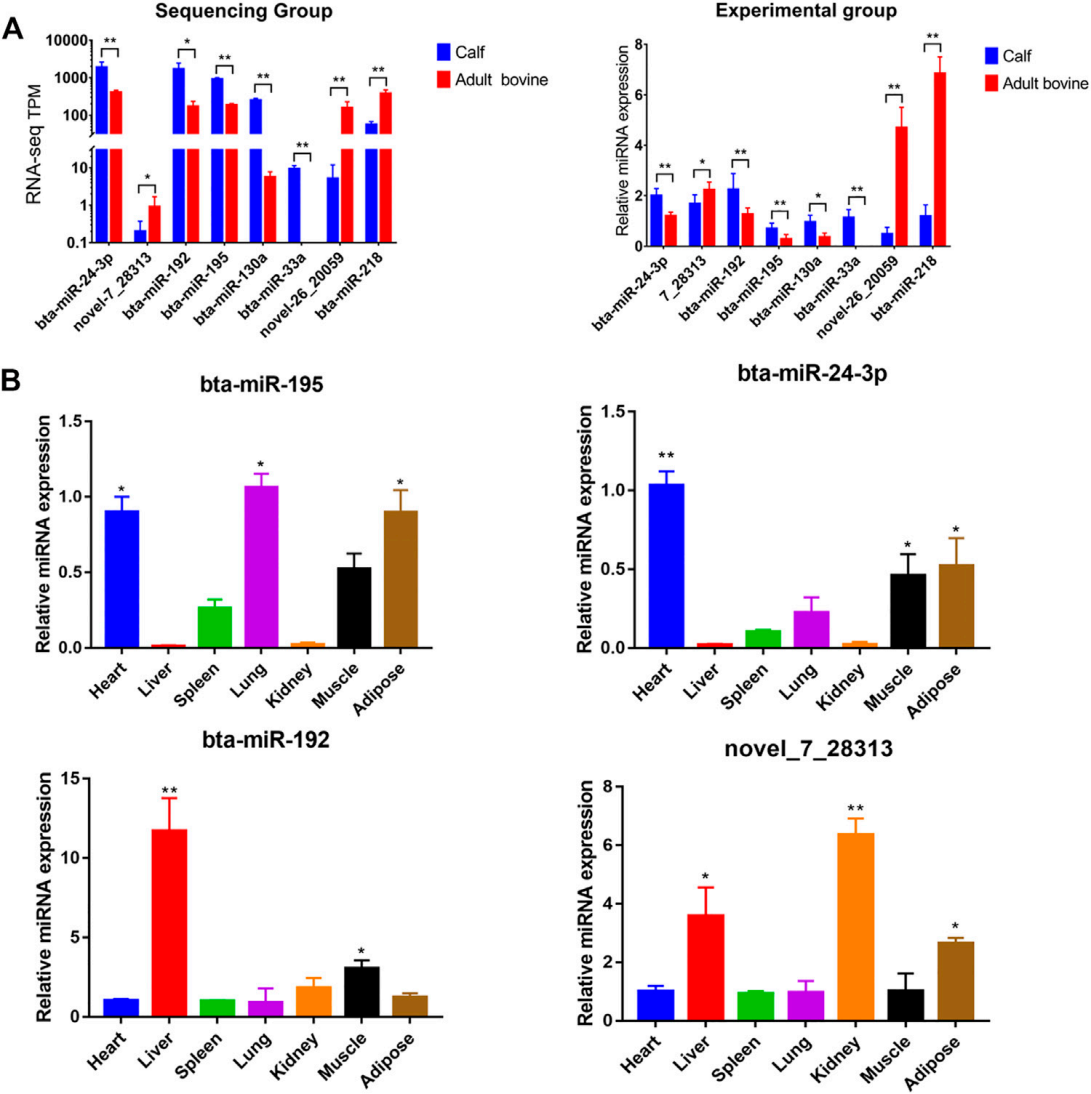
Validation of sequencing accuracy and expression profiles of key miRNAs. **(A)** Expression of miRNAs by RNA-seq **(left)** and qPCR **(right)** at different developmental stages, and the color “blue” and “red” represents calf and adult Xinyang buffalo, respectively; **(B)** Relative expression profile of four interested miRNAs (*bta-miR-195*, *Top left*; *bta-miR-24-3p*, top right; *bta-miR-192*, bottom left, *novel_7_28313*, bottom right) in heart, liver, spleen, lung, kidney, muscle, and adipose tissue (mean ± SD, *n* = 3). The symbol “*”, “**”, and “***” represent *p* < 0.05, *p* < 0.01, and *p* < 0.001, respectively.

To validate the accurancy of the sequencing data, eight miRNAs (*bta-miR-192*, *bta-miR-195*, *bta-miR-24-3p*, *bta-miR-30a*, *bta-miR-130a*, *bta-miR-218*, *novel-miR-7_28313*, and *novel-miR-26_20059*) were randomly selected from the 282 DE miRNAs to identify their expression pattern at the two growth stages by qRT-PCR. All eight were differentially expressed across the two stages (*p* < 0.05) and the expression pattern was consistent across miRNA sequencing and qPCR (Figure 5A), showing that the expression values obtained from miRNA sequencing were reliable.

### Adipocyte Differentiation Induction and Bta-miR-195 Expression Analysis

**FIGURE 6:**
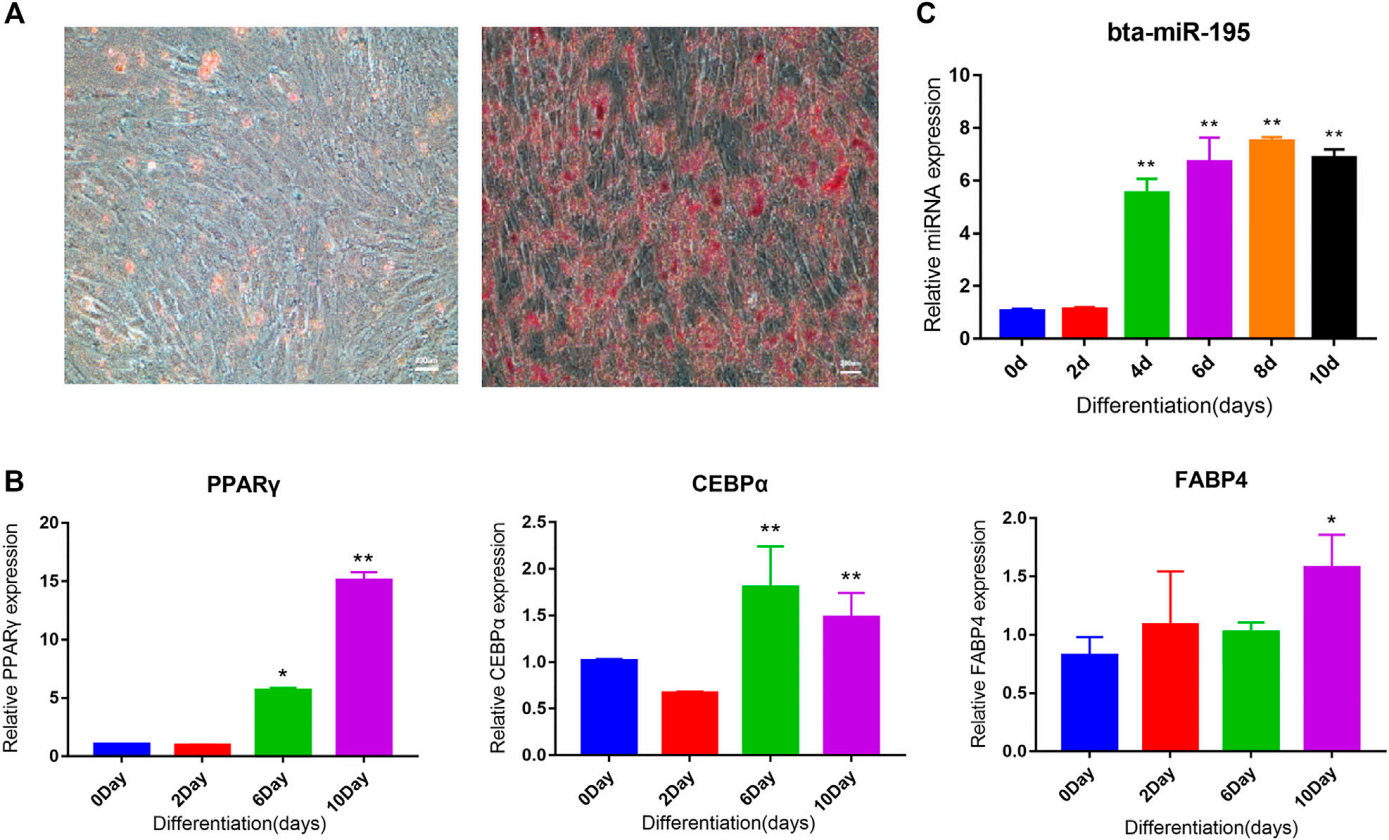
Adipocyte differentiation induction and bta-miR-195 expression analysis. **(A)** Oil red O staining of adipocytes 10 days after induction of bovine primary adipocytes differentiation, with the control group (right, 200X); **(B)** and **(C)** Real-time quantitative detection of *PPARγ*, *C/EBPα*, *FABP4*, and miR-195 markers of adipocyte differentiation. Gene expression plotted as fold-change relative to day 0 (mean ± SD, *n* = 3, the significance symbols are the same as Figure 5).

Analysis of the tissue expression profile of four miRNAs that could target fat function genes showed that bta-miR-195 was highly expressed in fat (Figure 5B). We selected bta-miR-195 for subsequent functional verification on adipocyte differentiation. To explore its expression pattern, primary adipocytes collected from subcutaneous adipose tissue were induced to differentiation. Oil red O staining showed that adipocytes induced for 10 days were stained while preadipocytes were not, indicating that the primary adipocytes were successfully induced to differentiated adipocytes (Figure 6A). *PPARγ*, *CEBPα*, and *FABP4*, as marker genes of adipocyte differentiation, were also upregulated, confirming the successful induction of differentiation (Figure 6B). Expression values of *bta-miR-195* at days 0, 2, 4, 6, and 10 of adipocyte differentiation were determined by qPCR. The expression of bta-mir-195 was significantly upregulated (*p* < 0.05) on day 4 of induction and maintained high expression during adipocyte differentiation until day 10 (Figure 6C), suggesting it may play an important role in the later stages of differentiation.

### Effects of Bta-miR-195 on Lipid Accumulation in Adipocytes

**FIGURE 7:**
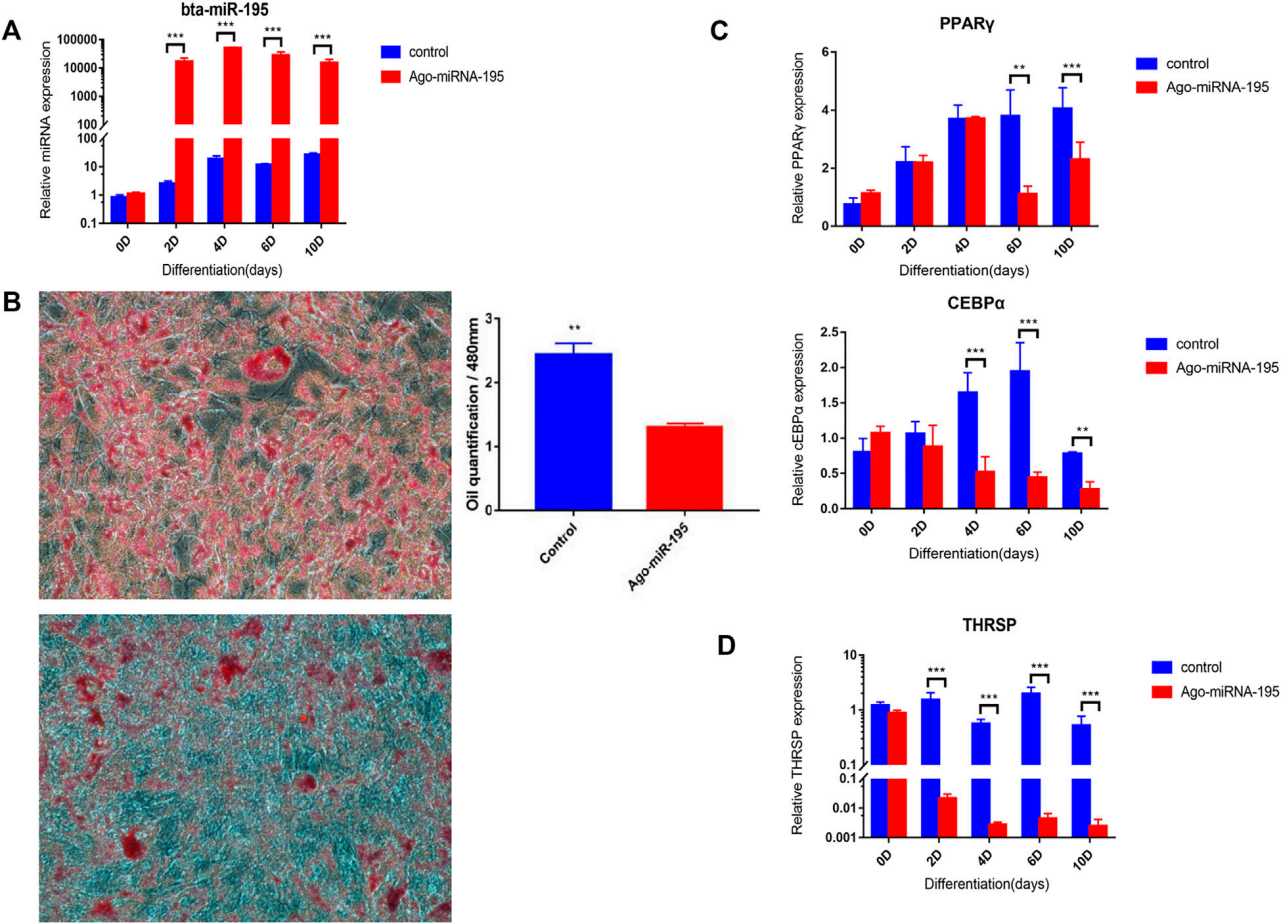
Analysis of the functional role of bta-miR-195 in fat deposits. **(A)** Transfection efficiency of *miR-195* agonist agomiR; **(B)** Quantitative Oil red O staining (200X) after 10 days of high expression of *miR-195*; **(C)** Expression of adipogenic marker genes in primary adipocytes after high expression of *miR-195*; **(D)** Expression of candidate target gene *THRSP* in primary adipocytes after high expression of *miR-195* (mean ± SD, *n* = 3). The significance symbols are the same as Figure 5.

Overexpression of bta-mir-195 was induced to further explore its effects on the adipogenic differentiation of bovine adipocytes. The transfection efficiency of *miR-195* was determined by qRT-PCR on days 0, 2, 4, 6, and 10 of induction. Expression of *bta-miR-195* increased significantly (*p* < 0.001) on day 2 of transfection and maintained high expression until day 10, indicating that the agomiR used was effective and stable and that transfection was successful (Figure 7A). The expression of lipogenic markers during adipocyte differentiation was quantified by qPCR, adipocytes were stained with Oil red O, and lipid accumulation was quantified using OD values after 10 days of induction. It was found that the expression of lipogenic marker genes *PPARγ* and *CEBPα* was significantly inhibited (*p* < 0.001) and the quantity of lipid decreased significantly with overexpression of bta-mir-195 (Figure 7B), indicating the negative regulatory effect of *bta-miR-195* on adipocyte differentiation (Figure 7C). The expression of *THRSP*, a predicted target gene of *bta-miR-195*, was significantly reduced (*p* < 0.001) after day 2 of transfection (Figure 7D), which was contrary to the overexpression of *bta-miR-195.*

### *Bta-miR-195* Inhibition of Lipid Accumulation by Targeting *THRSP*

**FIGURE 8:**
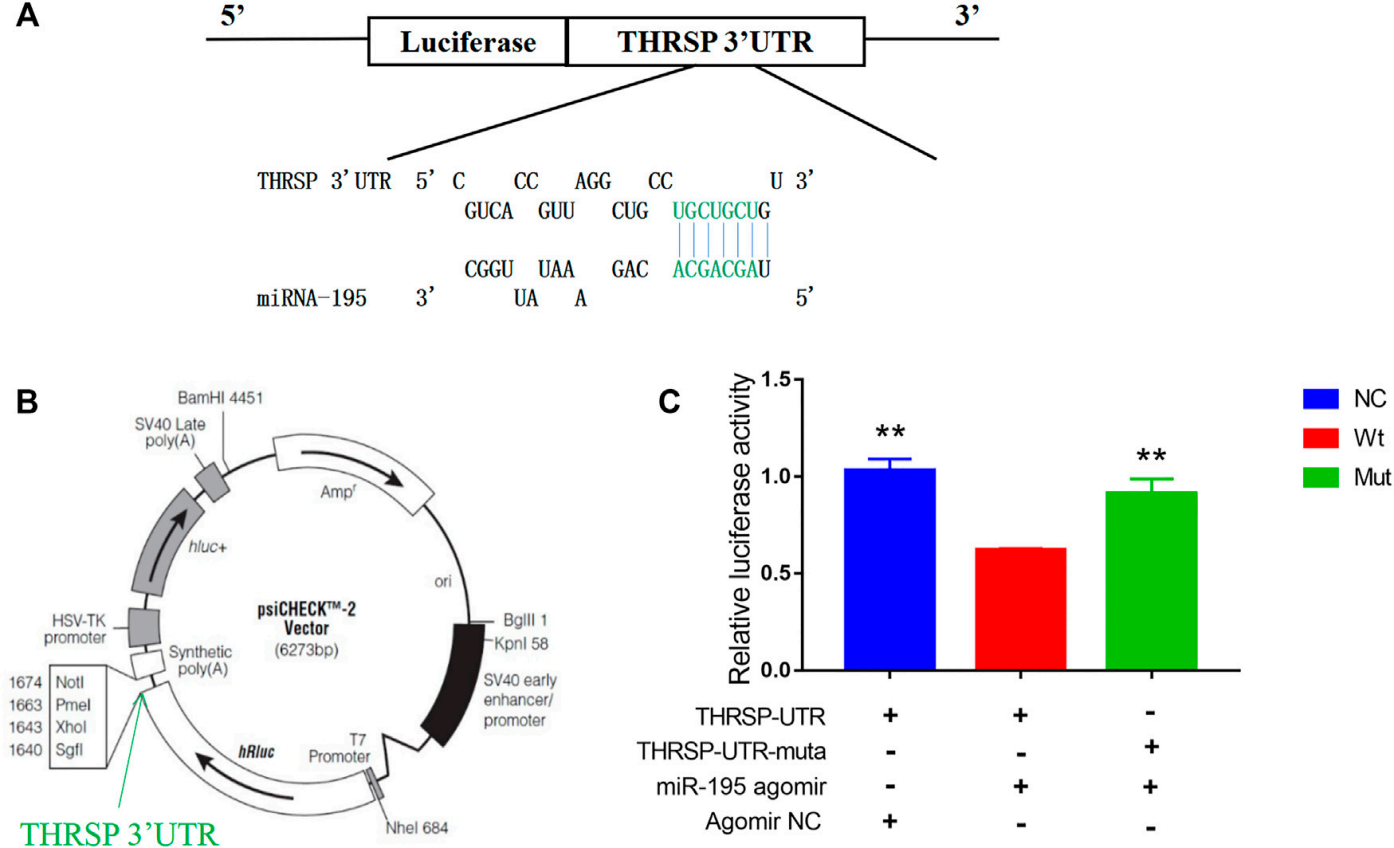
Dual-luciferase reporter assay for *THRSP*-targeting by *bta-miR-195*. **(A)** AgomiR pairing schematic of *miR-195* and *THRSP* 3′UTR. Nucleotides in green represent the “seed agomiR sequence” of *miR-195*, with mutation nucleotides in turquoise. **(B)** psiCHECK-2 agomiR vector map with the insertion site of *THRSP* 3′UTR marked. **(C)** *THRSP*-3′ UTR and its agomiR mutation luciferase reporter vectors co-transfected with *miR-195* agomiR (or negative agomiR control) into 293T cells. Dual-luciferase assay performed 48 h after transfection. Results expressed as relative luciferase activity (mean ± SD, *n* = 6, ANOVA, the significance symbols are the same as Figure 5).

It was predicted that *THRSP* was a target gene of *bta-mir-195*. A dual-luciferase reporter assay was carried out to detect the targeting and binding of *bta-mir-195* to *THRSP.* The fluorescence of *bta-mir-195* agromiR was significantly lower than in the NC and mutant groups (*p* < 0.001). Fluorescence in the mutant vector group was lower than in NC, but significantly higher than in the wild vector group (*p* < 0.01). This may be due to excess inserts in the mutant vector affecting the transcriptional activity of *bta-mir-195*. These results indicate that *bta-mir-195* targeted *THRSP* 3′UTR and inhibited the expression of *THRSP* (Figure 8).

## Discussion

Buffalo breeds in China are plentiful and have high meat products, which can be considered as an essential source of meat (Sun et al., 2020). However, its rough muscle fibers and low IMF abundance have resulted in a meager market share. Fat deposition abundance is one of the major determinants of meat tenderness and juiciness. Still, the dorsal subcutaneous fat above the longest dorsal muscle can reflect the effect of IMF deposition. The measurement of dorsal fat thickness (dorsal subcutaneous fat thickness) is used to predict intramuscular fat deposition *in vivo*, as shown in pig (Monziols et al., 2006; Knecht and Duziński, 2016), cattle (Nogalski et al., 2017), sheep (Chay-Canul et al., 2016), providing reference to study intramuscular fat deposition in buffalo. Fat deposition is regulated by many small molecules, such as lncRNAs and miRNAs. Still, there exists a shortage of studies on related miRNAs and their regulatory mechanisms in the development of buffalo fat deposition. Therefore, the transcriptome sequencing of Xinyang buffalo at different developmental stages was performed in the hope of identifying miRNAs and exploring their molecular mechanisms associated with fat deposition.

As expected, we identified several miRNAs associated with adipogenesis, such as *let-7*, *miR-10b*, *miR-148a*, *miR-27b*, *miR-29b*, and *miR-195*. It has been shown that the *let-7* miRNAs family was highly expressed in adipose tissue and that family members *let-7f*, *let-7e* could target *HMGA2* and *MMP9*, key genes for lipogenesis, and thus affect triglyceride synthesis and lipid metabolism (Sun et al., 2009; Ventayol et al., 2014). The expression of *miR-10b* was negatively correlated with the lipogenic marker genes *CEBPα*, *PPARγ* and *AP2*, and *miR-10b* could play a key regulatory role in hADSC lipogenic differentiation by inhibiting *SMAD2* gene expression and consequently affecting the TGF-β pathway (Li et al., 2018). During adipogenic differentiation of ADSCs, upregulation of *miR-29b* could promote adipogenesis by enhancing *SP1* indirectly targeting *TNF-α* (Li et al., 2018). The up-regulation of these miRNAs may indirectly target fat deposition-related pathways and proteins by affecting the translation of their target genes, which could finally regulate fat deposition and improve buffalo meat quality.

Considering that some important miRNAs (miRNAs involved in fat deposition but not significantly differential expression) may be lost by only significant expression of miRNAs, we further used WGCNA to uncover important miRNAs associated with fat deposition and found that *miR-93*, *miR-199a-3p*, and *miR-195* were closely associated with adipogenesis development. It was shown (Li et al., 2018) that decreased *miR-93* expression increased lipid droplet content, and *miR-93* restricted early adipocyte precursor self-renewal by targeting *Tbx3*. Meanwhile, *miR-93* targeted *Sirt7* to induce precursor adipocyte differentiation and maturation and affect lipid redox. *miR-199a-3p* overexpression attenuated lipid accumulation and adipogenic gene expression and impaired brown adipocyte metabolism by reducing mitochondrial DNA content and respiration (Gao et al., 2018).

In addition, to further elucidate the mechanisms associated with adipogenesis regulation by these miRNAs (*miR-195*, *miR-192*, *miR-7_28313*, *miR-24-3p*), we focused on this on the functional enrichment of target genes of key miRNAs. Excitingly, these target genes were indeed enriched in pathways related to adipogenesis, such as fatty acid metabolism (Mika et al., 2019), PPAR signaling pathway (Fanale et al., 2017). Not only could these miRNAs regulate the expression of target genes to participate in the adipogenic process, but these miRNAs may be collectively involved in several comprehensive signaling pathways affecting fat deposition.

In this study, we finally focused *miR-195* according to differential analysis and co-expression network analysis. Expression features showed a high abundance of *miR-195* expression in adipose and a significant dynamic expression of *miR-195* with the maturation of adipocyte differentiation, suggesting a possible regulatory role in adipose differentiation; this was confirmed by its overexpression. The marker of fat deposition is the differentiation of many adipocytes, and this process is the accumulation of lipid droplets after the differentiation of primary adipocytes (Moreno-Navarrete and Fernández-Real, 2017). During the differentiation of primary adipocytes, the cells first change from the shuttle to round shape and begin to differentiate and gradually produce fine lipid droplets, which accumulate and fuse with the time of differentiation and eventually form large lipid droplets that can be stained. The ability of primary adipocytes to be induced into a round shape by inducers is indicated by the upregulation of the lipogenic marker gene. The eventual inability to form large lipid droplets under the sustained effect of overexpressed *miR-195* suggests that it can inhibit adipocyte differentiation and thus affect fat deposition. It has been reported that the ectopic expression of *miR-195* suppresses the expression of *INSR*, thereby impairing the insulin signaling cascade and glycogen synthesis in HepG2 cells (Yang et al., 2014). Meanwhile, *miR-195* is able to target *FNAS* to regulate ATP consumption and cell migration (Mao et al., 2012; Lin et al., 2021). This suggests that the same miRNA will serve different functions depending on the target genes. In our study, *miR-195* affected lipid droplet formation by targeting *THRSP*. Although there was a temporary differentiation in the pre-lipogenesis-induced differentiation phase, *THRSP* was significantly suppressed all the time with the continuous overexpression of miRNAs, thus inhibiting lipid droplet formation.

**FIGURE 9:**
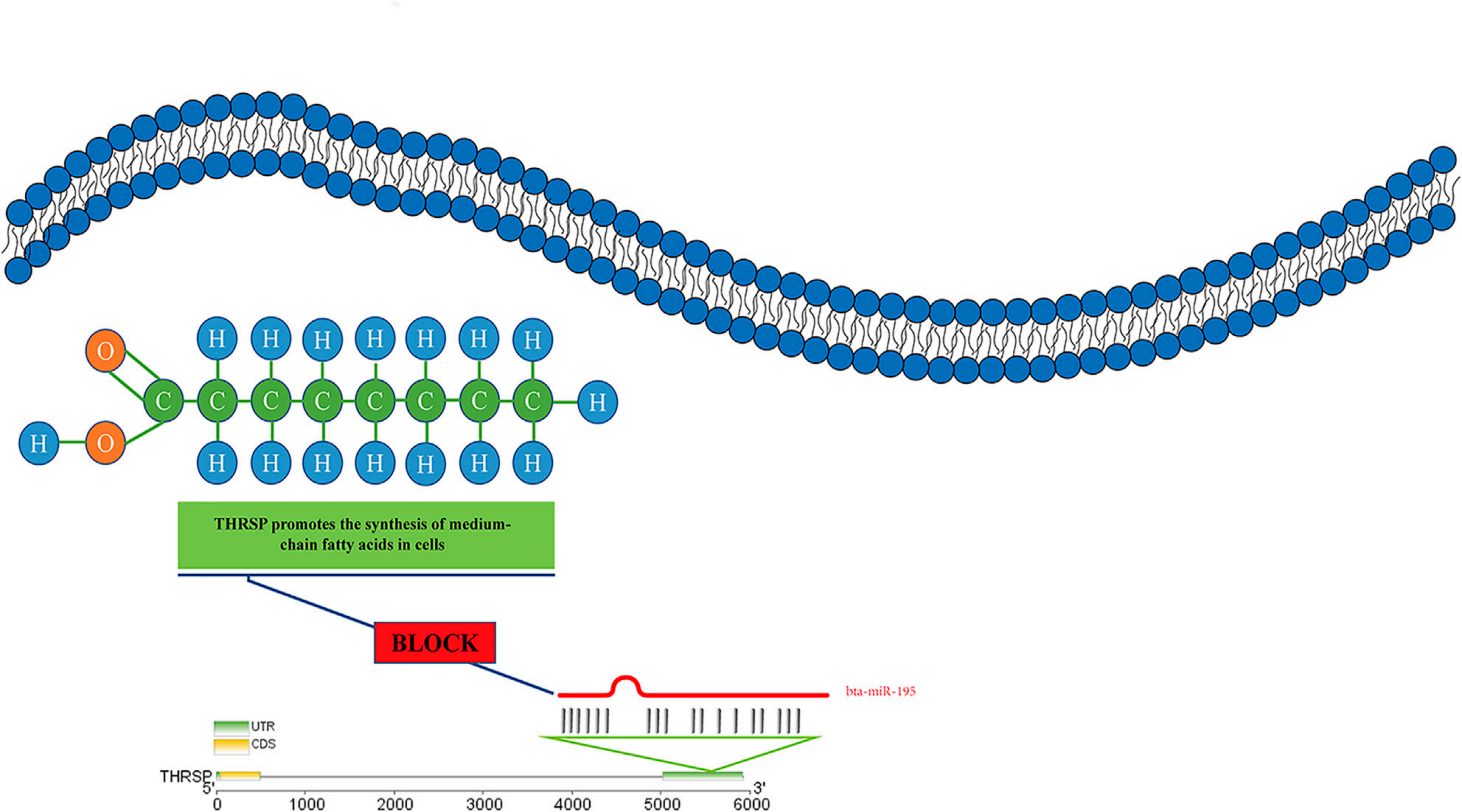
*miR-195* inhibits medium-chain fatty acid synthesis by targeting *THRSP*.

*THRSP* is a small nuclear protein acting as a key lipogenic activator and is activated by thyroid hormone triiodothyronine (T3), carbohydrates, glucose, and insulin (Wu et al., 2012). *THRSP* is abundant in lipogenic tissues such as fat, liver, and mammary gland (Chou et al., 2007; Ren et al., 2017). Many studies show that *THRSP* plays a vital role in regulating lipogenesis in goat mammary epithelial cells by increasing the concentrations of C12:0, C14:0 and synthesizing medium-chain fatty acids *in vitro* (Yao et al., 2016). In mice, *THRSP* acts in the regulation of diet-induced obesity, while *THRSP*-knockout leads to deficiencies in *de novo* lipogenesis in the lactating mammary gland (Yao et al., 2016). T*HRSP* is a potential molecular marker for fat deposition in cattle since its mRNA abundance was shown to be elevated in skeletal muscle with high IMF concentration (Schering et al., 2017). These results suggest that *miR-195* may inhibit the synthesis of medium-chain fatty acids by inhibiting *THRSP*, thus leading to the failure of lipid accumulation (Figure 9). In addition, the comparison showed that the *THRSP* 3′UTR binding sites were highly consistent, indicating that *miR-195* had the same function in cattle and buffalo (Supplementary Figure S3). Therefore, *bta-miR-195* plays a significant role in inhibiting lipogenesis by suppressing the expression of *THRSP*, and knockdown of *bta-miR-195* may positively affect lipid deposition.

While this study validated miRNAs associated with fat deposition from different perspectives, it still leaves something to be desired. We were unable to isolate any primary adipocytes from the longest muscle of the buffalo dorsum due to poor fat deposition in Xinyang buffalo (Huang et al., 2020), so we adopted samples obtained from the subcutaneous fat of the dorsum for small RNA sequencing. Therefore, our experiments were just able to reflect the results of intramuscular fat deposition in cattle from the side. Second, we should use public database resources to validate the expression of candidate miRNAs in other bovine species to confirm the accuracy of our results.

Fat deposition is regulated by some miRNAs, which inhibit adipocyte differentiation and hinder adipocyte formation by regulating their target genes, resulting in less intramuscular fat deposition and inferior meat quality in Xinyang buffalo, but this result needs more functional experiments to prove. This study used gene sequencing technology to systematically and comprehensively identify miRNAs that regulate adipogenesis in buffalo and explore their regulatory mechanisms, providing a theoretical basis for enriching muscle fat deposition in buffalo. In addition, a miRNA (*miR-195*), which may be related to adipose function, was screened, and its mechanism of action in adipocytes was elucidated, which laterally confirmed the function of the screened miRNAs in the adipose deposition. This study enriches the theory of fat deposition in buffalo and provides a theoretical basis for the selection and breeding of buffalo for meat.

## Data Availability

The datasets presented in this study can be found in online repositories. The names of the repository/repositories and accession number(s) can be found below: GSE171146, GSE119064, GSE112744.
