# Supplementary figures and images for: Identification and Functional Verification Reveals that miR-195 Inhibiting THRSP to Affect Fat Deposition in Xinyang Buffalo
Source: Front Genet. 2021 Dec 22;12:736441. doi: 10.3389/fgene.2021.736441 (PMC8727870; doi:10.3389/fgene.2021.736441)

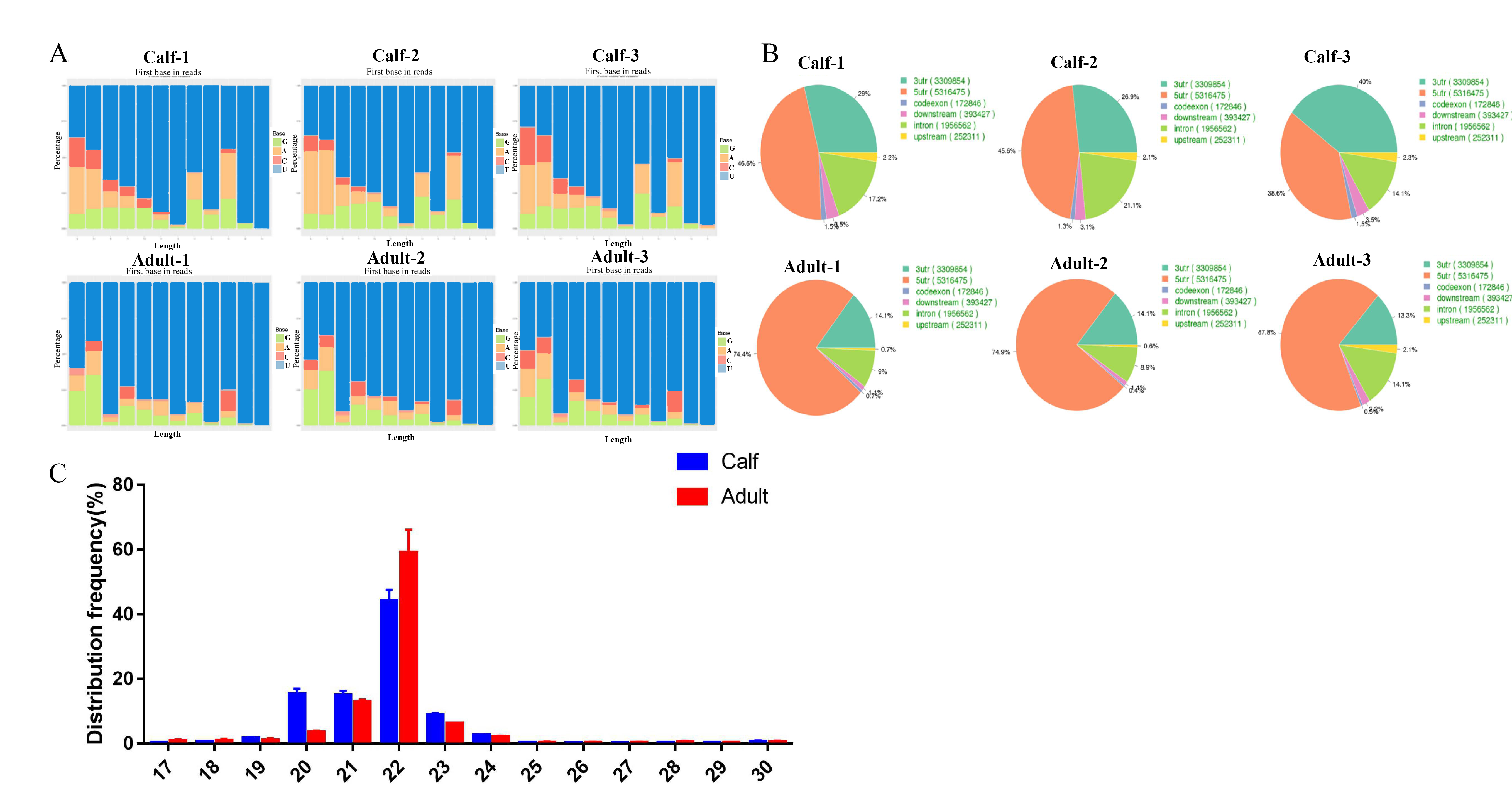

Supplement: Supplementary file 3 [file Image1.JPEG]

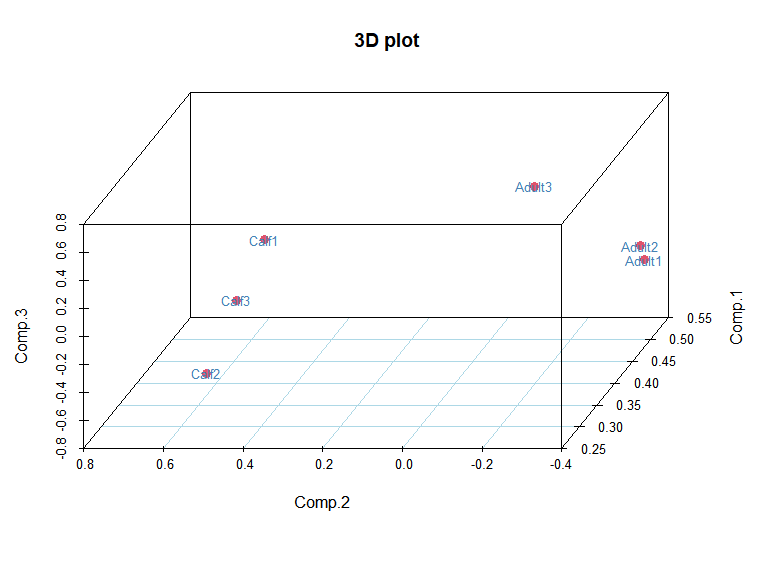

Supplement: Supplementary file 7 [file Image2.TIFF]

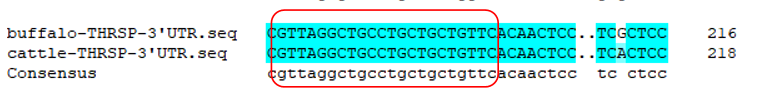

Supplement: Supplementary file 8 [file Image3.PNG]
